# Inpatient Palliative Care for Pediatric Neurologic and Neuromuscular Illnesses in the U.S.—A National Perspective

**DOI:** 10.3390/children13081072

**Published:** 2026-08-13

**Authors:** Zahra Mojtahedi, Pearl C. Kim, Yonsu Kim, Ji W. Yoo, Jay J. Shen

**Affiliations:** 1 Research Capacity Core (RCC), Northern Arizona University, Flagstaff, AZ 86011, USA; zahra.mojtahedi@nau.edu; 2 Department of Healthcare Administration and Policy, School of Public Health, University of Nevada, Las Vegas, NV 89119, USA; pearl.kim@unlv.edu (P.C.K.); yonsu.kim@unlv.edu (Y.K.); 3 Department of Internal Medicine, Kirk Kerkorian School of Medicine, University of Nevada, Las Vegas, NV 89106, USA; ji.yoo@unlv.edu

**Keywords:** pediatric, palliative care, neurologic, neuromuscular, disability, inpatient

## Abstract

**Highlights:**

**What are the main findings?**

- Palliative care utilization of children with neurologic and neuromuscular illnesses remains low.
- Disparities in receipt of palliative care exist across sociodemographic factors such as race/ethnicity and income.

**What is the implication of the main finding?**

- Improving awareness of palliative care among pediatric care providers is important for them in offering palliative care as a care option for children with life-limiting illness such as neurologic and neuromuscular illnesses.
- Integrating palliative care into pediatric practice is likely to enhance outcomes of quality of life for children with life-limiting illnesses.

**Abstract:**

Background: Pediatric neurologic and neuromuscular illnesses, often progressive and debilitating, require comprehensive medical management focused on enhancing quality of life. Despite the benefits of palliative care in managing symptoms and providing psychological support, there is limited knowledge on its associated factors and utilization for pediatric neurologic and neuromuscular illnesses in hospital settings. Methods: This study analyzed the National Inpatient Sample (NIS) database, covering hospitalizations of children under 18 years with neurologic and neuromuscular diagnoses from 2016 to 2020, to examine associations of palliative care with sociodemographic, clinical, and hospital factors. Results: Out of 139,598 pediatric patients, 4273 (3.1%) received palliative care. Palliative care recipients were younger (mean age 5.3 vs. 6.4 years), had more diagnoses (17.8 vs. 10.9) and procedures (4.0 vs. 2.4), higher illness severity scores (3.4 vs. 2.6), longer LOS (18.7 vs. 9.9 days), and higher hospital charges ($332,559 vs. $150,727). In-hospital mortality was significantly higher among palliative care recipients (32.9% vs. 2.4%). The utilization of palliative care increased annually (OR = 1.08, 95% CI [1.05, 1.11]). Black patients had lower odds (OR = 0.90, 95% CI [0.81, 1.00]). Higher severity scores (OR = 2.09, 95% CI [1.99, 2.20]) were positively associated with palliative care. Smaller hospitals and urban nonteaching hospitals were inversely associated with palliative care utilization. Conclusions: Palliative care utilization for pediatric neurologic and neuromuscular illnesses remains low, with significant disparities based on sociodemographic and hospital-related factors. Enhanced awareness and integration of palliative care in pediatric practice, especially in underutilized settings, are crucial for improving outcomes and quality of life for these patients.

## 1. Introduction

Pediatric neurologic and neuromuscular illnesses pose significant challenges in medical management since they are often progressive, leading to a continuous decline in health and mobility [1,2]. The course of these conditions necessitates a multidimensional approach to care, emphasizing not only medical treatment but also the enhancement of patient quality of life [3]. Palliative care can significantly address pain management, symptom control, and psychological support for children with these illnesses and their families and caregivers, but it is often underutilized in pediatric settings [3,4,5].

Palliative care, which is a specialized care model, can integrate comfort care with other treatments and is delivered by an interdisciplinary team of healthcare professionals in different settings such as hospitals and homes [4,6,7]. The team works collaboratively to tailor interventions that meet the unique needs of each patient, aiming to maintain as much comfort and functionality as possible throughout the course of the disease [4,6,7]. However, despite its benefits, the implementation of inpatient palliative care varies widely across different regions and institutions, influenced by sociodemographic, clinical, and hospital factors [8,9].

Pediatric neurologic and neuromuscular illnesses encompass a spectrum of conditions affecting the nervous system and muscles in children, presenting complex challenges for long-term management [4,10]. From cerebral palsy to muscular dystrophy, these conditions can have profound effects on the development, mobility, and quality of life of children [4,10]. Management of these illnesses often requires a multidisciplinary approach involving pediatric neurologists, geneticists, rehabilitation specialists, physical therapists, and occupational therapists [9]. Incorporating a palliative care team has attracted much attention in recent years. These conditions are often associated with lifelong disabilities and limitations, necessitating additional comfort care [4,10]. Palliative care plays a crucial role in addressing the physical, emotional, and psychosocial needs of both the child and their family, focusing on symptom management, comfort, and enhancing overall well-being [9]. However, parents have shown reluctance to accept palliative care for their children, largely due to the misconception that it is exclusively meant for patients at the end of life [9,11]. The low utilization rate of pediatric palliative care limits conducting quantitative research in this area [5,9,12,13]. Moreover, empirical research on the utilization of inpatient pediatric palliative care remains limited. There is a need for further investigation using large national datasets to gain deeper insights into the factors associated with the utilization of pediatric palliative care, which is crucial for enhancing pediatric palliative care services.

The purpose of this study was to investigate the overall trend of palliative care utilization and the association of sociodemographic factors with inpatient palliative care for children with neurologic and neuromuscular illnesses on a national scale. Specifically, this study aimed to address what socioeconomic factors are associated with: (1) use of inpatient palliative care, (2) number of comorbidities, (3) length of hospital stay, and (4) total hospital charges. This investigation sheds light on current clinical practices and potential areas for policy enhancement, trying to improve the standard of care for this vulnerable patient population.

## 2. Methods

### 2.1. Study Design and Data Source

This study was a retrospective, pooled cross-sectional analysis and was conducted using de-identified discharge data obtained from the National Inpatient Sample (NIS) database. The unit of analysis was hospital discharge. The NIS, part of the Healthcare Cost and Utilization Project (HCUP), captures data from over 7–8 million hospital stays each year, constituting a 20% stratified sample of hospitals across 44–49 states (varied by year) in the U.S. [7]. The data was accessed following the completion of a data use agreement with the Agency for Healthcare Research and Quality (AHRQ), which sponsors HCUP. Detailed stratified sampling approach is provided by AHRQ [7]. The Institutional Review Board at the University of Nevada, Las Vegas, reviewed and approved this study. The study included children younger than 18 years old who were hospitalized between 2016 and 2020 with a diagnosis of brain and spinal cord malformations, mental degradation, CNS degeneration and diseases, infantile cerebral palsy, epilepsy, other disorders of CNS, occlusion of cerebral arteries, muscular dystrophies and myopathies, and movement diseases [14]. The diagnoses were identified through specific International Classification of Diseases, Tenth Revision, Clinical Modification (ICD-10-CM) codes (Supplementary File S1).

### 2.2. Measures

Five outcomes or response variables included the receipt of palliative care consultation, the numbers of diagnoses, the numbers of procedures, LOS and total hospital charges among pediatric, neurologic, and neuromuscular hospitalizations. Independent variables included sociodemographic characteristics such as sex (male and female (as reference)), age, race/ethnicity (White (as reference), Black, Hispanic, Asian, and Native American), insurance type (private insurance (as reference), Medicaid, self-pay, and free care), and median household income by the patient’s ZIP code (0th to 25th percentile, 26th to 50th percentile, 51st to 75th percentile, and 76th to 100th percentile (as reference)). Since age was mostly used as a control variable in the analysis, we did not exclude any age group, including children younger than one year old. Clinical data included illness severity as classified by the All-Patient Refined Diagnosis-Related Groups (APRDRG) with severity levels ranging from 1 (minor) to 4 (extreme), I10_NDX (indicates the total number of ICD-10-CM diagnoses coded on discharge), I10_NPR (indicates the total number of ICD-10-CM procedures coded on the discharge), in-hospital death, hospital length of stay (LOS), and total hospital charges, with the charges adjusted for annual healthcare inflation estimated by the Center for Medicare and Medicaid Services (CMS) [15]. Hospital-related variables, categorized by AHRQ [16], included hospital bed size (large, small, medium), and hospital status (urban teaching hospital, urban nonteaching hospital, rural hospital). The presence of palliative care services was identified through specific codes of Z51.5 for ICD-10-CM.

### 2.3. Statistical Analysis

Weighted multivariable regression models were used to examine factors associated with the five outcomes. The sampling weight is calculated by the HCUP program and available in the NIS dataset, which can be used to calculate the national estimate [7]. Factors included sex (reference: male), age, race/ethnicity (White (reference), Black, Hispanic, Asian and Pacific Islander, Native American, other), insurance type (Medicaid, private insurance, uninsured, other), quartile of patient median household income by ZIP code (reference: the 76–100% quartile), APRDRG (Levels 1–4), hospital bed size (small, medium, and large (reference)), and hospital status. All models were adjusted for the U.S. Census divisions for potential geographic variations. A missing category was created for each variable and included in the multivariable analysis to preserve the sample size; however, results for the missing-value category were not reported. Odds ratios (ORs) and 95% confidence intervals (CIs) for logistic models and β-coefficients and standard errors for linear models were estimated using SAS statistical software version 9.4. All *p*-values were two-tailed, with values less than 0.05 deemed to indicate statistical significance.

## 3. Results

Table 1 displays the characteristics of inpatient palliative care for pediatric neurologic and neuromuscular hospitalizations, and the proportion of patients receiving palliative care was 3.1%, 2.8%, 2.9%, 3.0%, and 3.7% for the years 2016 to 2020, respectively, with a five-year average of 3.1%. The majority of patients, both those who received palliative care and those who did not, were male, with percentages close to 53%. Regarding race/ethnicity, the percentage for White, Black, Hispanic, Asian, and Native American hospitalizations receiving palliative care were 48.3%, 18.0%, 22.4%, 3.8%, and 1.0%, respectively, compared to those who did not (50.7%, 17.0%, 21.9%, 3.5%, and 0.9%, respectively). A larger proportion of palliative care recipients compared to non-recipients had private insurance (61.0% vs. 57.2%) and self-pay (5.3% vs. 1.6%). The mean age of patients who received palliative care was lower at 5.3 years compared to 6.4 years for those who did not. Patients who received palliative care also had a higher average number of diagnoses (17.8) and procedures (4.0) compared to those who did not (10.9 diagnoses and 2.4 procedures). Illness severity, indicated by the APR-DRG severity score, was higher among palliative care patients (mean score 3.4) compared to non-palliative care patients (mean score 2.6). Additionally, the average LOS was longer for patients who received palliative care (18.7 days) compared to those who did not (9.9 days), as were the total hospital charges, with an average of $332,559 for palliative care patients compared to $150,727 for non-palliative care patients. In-hospital mortality was significantly higher among patients who received palliative care (32.9%) compared to those who did not (2.4%). Large urban hospitals were the most common setting for both groups, but there was a higher percentage of patients receiving palliative care in large hospitals (63.5%) compared to those who did not (57.9%).

Table 2 shows the results of multivariable analysis. There was an association between the year of admission and palliative care utilization, with each subsequent year showing an increase in the odds of utilization (OR = 1.08, 95% CI [1.05, 1.11]). Older pediatric patients had lower odds of utilizing palliative care services (OR = 0.98, 95% CI [0.97, 0.98]). Females had higher odds of palliative care utilization compared to males (OR = 1.10, 95% CI [1.03, 1.18]). Black patients showed decreased odds of utilizing palliative care (OR = 0.90, 95% CI [0.81, 1.00]). Severity of illness, as indicated by APR-DRG severity scores, exhibited a strong association with palliative care utilization, with higher severity scores correlating with substantially increased odds of utilization (OR = 2.09, 95% CI [1.99, 2.20]. Patients from ZIP codes in the 26th to 50th income percentile demonstrated higher odds of utilizing palliative care (OR = 1.16, 95% CI [1.04, 1.29]). Hospitals with smaller bed sizes were associated with decreased odds of palliative care utilization (Small: OR = 0.77, 95% CI [0.70, 0.85]; Medium: OR = 0.82, 95% CI [0.75, 0.90]). Moreover, hospitals with patients admitted to urban, nonteaching hospitals had lower odds of palliative care utilization compared to those in urban, teaching hospitals (OR = 0.54, 95% CI [0.42, 0.69].

Table 3 demonstrates factors with relation to the total numbers of diagnoses and procedures among pediatric patients with neurological and neuromuscular disorders from 2016 to 2020 in U.S. hospitals. For the number of diagnoses, the yearly data showed a consistent increase in diagnosis counts over time with a regression parameter β-coefficient of 0.58. Patients receiving palliative care had a higher number of diagnoses, with a β-coefficient of 3.42. Age was also a significant factor, with each additional year increasing diagnosis counts by about 0.02. Females had fewer diagnoses than males, indicated by a β-coefficient of −0.08. Racial/ethnic variables showed that Black and Asian patients had more diagnoses compared to Whites, with a β-coefficient of 0.34 and 0.24, respectively. Insurance status impacted diagnosis counts; Medicaid and self-pay hospitalizations had more diagnoses, with estimates of 0.62 and 0.51, respectively. APR-DRG severity level was significantly associated with higher diagnosis counts, with a β-coefficient of 4.24. Patients from lower-income ZIP codes all had significantly more diagnoses. Hospital bed size with small and medium (vs. large) had significantly lower (β-coefficient: −0.28) and higher (β-coefficient: 0.16) diagnosis counts, respectively. Rural and urban, nonteaching hospitals (vs. urban, teaching) documented fewer diagnoses (β-coefficient: −1.22 and −0.40, respectively).

With regard to the number of procedures, the yearly increase in procedures was also significant, with a β-coefficient of 0.09. Receipt of palliative care was not significantly associated with number of procedures (β = 0.05, *p* = 0.50). Age inversely affects procedure counts, decreasing with each additional year by about −0.04. Females had fewer procedures than males, with a β-coefficient of −0.084. Black and Asian patients had more procedures than Whites, with β-coefficients of 0.18 and 0.18, respectively, whereas Native Americans had significantly fewer procedures, with a β-coefficient of −0.22. Insurance status reveals that Medicaid and free-care patients had fewer procedures than those with private insurance, with β-coefficients of −0.27 and −0.25. Clinical severity, as measured by APR-DRG, was strongly associated with an increased number of procedures, with a β-coefficient of 1.157. Patients in small and medium-sized hospitals (vs. large) had more procedures than those in large hospitals. Rural and urban nonteaching hospitals showed a significant decrease in procedures compared to urban teaching hospitals, with β-coefficients of −0.96 and −0.43, respectively (Table 3).

Table 4 lists factors with relation to hospital LOS and total charges among pediatric patients with neurological and neuromuscular disorders from 2016 to 2020 in U.S. hospitals. As for LOS, the annual trend showed an increase in LOS each year, with an average of 0.51 days. Palliative care was strongly associated with a longer LOS, showing a 4.03-day longer stay. Age was inversely associated with LOS, with an average of 0.62 days shorter for each additional year. Black and Asian patients had longer LOS compared to Whites, with 2.32 and 1.50 days longer, respectively. Medicaid, uninsured, and free-care patients all had shorter LOS compared to those with private insurance (0.38, 1.21, and 1.17 days shorter, respectively). Clinical severity, measured by APR-DRG, was positively associated with LOS with 7.38 days longer as severity increases by one level. Economic status, as indicated by ZIP code median income, showed that patients living in lower-income areas tended to have longer LOS. Patients in rural hospitals stayed 2.11 days shorter compared to their counterparts in urban teaching hospitals. Conversely, patients treated in urban nonteaching hospitals stayed 1.85 days longer (Table 4).

In terms of the total charges, yearly increases in hospital charges showed a rise of approximately $9212 each year. Palliative care was associated with higher hospital charges, adding about $57,000. Age showed an inverse association with charges, decreasing by $6873 for each additional year (*p* < 0.0001). Black, Hispanic, and Asian patients incurred significantly higher hospital charges than White patients ($28,226, $13,731, and $19,965 higher, respectively), whereas charges for Native American patients were slightly lower. Medicaid and free-care patients had significantly lower charges compared to those with private insurance by $18,495 and $24,834, respectively. Uninsured patients showed no significant difference in charges compared to those with private insurance. The severity of the condition, as measured by APR-DRG, was associated with an increase of $122,122. Median household income showed no significant association with hospital charges across different income quartiles. Medium-sized hospitals incurred significantly higher charges than large hospitals, with an increase of $17,763. Patients treated at rural hospitals incurred significantly lower charges—about $48,953 less on average—than those treated at urban teaching hospitals (Table 4).

## 4. Discussion

Our findings indicate an overall rising trend in the use of inpatient palliative care services among pediatric patients with neurologic and neuromuscular conditions from 2017 to 2020 (2016 has a relatively high number for some reason). This increase likely reflects both expanded availability and greater awareness among clinicians and families of the role palliative care plays in improving quality of life for children with complex medical conditions [17]. Additionally, improvements in care delivery models and greater integration of palliative care into standard clinical practice may have driven this trend [18]. Despite these gains, utilization remains substantially lower than in adult populations [7,19,20]. The palliative care utilization rate reported from the previous inpatient pediatric cancer cohort was similar to that of our study [21]. One key explanation, as our findings suggest, is that palliative care is still predominantly offered to patients nearing end of life, a pattern that aligns with prior studies [22,23].

The utilization of palliative care services was also associated with sociodemographic factors. Older pediatric patients had lower odds of utilizing palliative care services, suggesting potential more severe conditions existing among younger patients especially those younger than one year old, which remits future research. Females demonstrated higher odds of palliative care utilization compared to males, which is consistent with that in adult patients [7,19,20]. Race/ethnicity and health insurance were not associated with palliative care utilization, which was in contrast to what was usually reported in adult patients [7,19,20,24]. Clinical factors may play a more dominant role in making treatment decisions for pediatric patients, potentially outweighing the influence of race and ethnicity and thereby attenuating the disparities more commonly reported in adult populations, as our findings indicate that APR-DRG severity scores show strong positive association with use of palliative care and are also consistent with prior studies on pediatric palliative care [25,26,27]. These findings are similar in adult patients [7,19,25], which confirms that children with higher severity scores are likely to experience greater symptom burden and require specialized care, such as palliative care, to manage their symptoms and enhance their quality of life. Combining the findings of race, health insurance status, and the severity of illness, it appears that clinical symptoms and conditions are stronger deciding factors than both race and health insurance for use of palliative care among pediatric patients with neurologic and neuromuscular illnesses.

In addition, hospital-related factors (bed size and teaching status) were also found to be significantly associated with the utilization of palliative care services. The main reason that medium-sized hospitals provide less palliative care than large hospitals is that the medium-sized hospitals may be large enough to care for children with medically complex conditions but may not have dedicated (pediatric) palliative care programs and trained palliative care providers. Hospitals with smaller bed sizes were associated with decreased odds of palliative care utilization, which might be related to the unavailability of these special services (e.g., no palliative care physicians and other providers) in small hospitals, consistent with past studies on bed size as a factor of inpatient palliative care [19,25,28]. Similarly, patients admitted to urban, nonteaching hospitals had lower odds of palliative care utilization compared to those in urban, teaching hospitals [29]. It is interesting that rural hospitals and urban teaching hospitals are comparable in terms of providing palliative care. No literature has studied this issue and future research is needed. One hypothesis that may be tested by future studies is that, in rural hospitals, there are often no dedicated pediatric palliative care teams, so general pediatricians or family physicians provide basic symptom management and end-of-life support themselves.

The numbers of diagnoses and procedures displayed similar, but sometimes different, associations with palliative care utilization, sociodemographic, clinical, and hospital-related factors. Patients under palliative care had a higher number of diagnoses, but not procedures, compared to those not receiving such care, indicating some more severe patients (e.g., with a high number of diagnoses) tend to receive palliative care that could be a care option for more intensive and advanced clinical procedures [2,30,31]. Small hospitals (vs. large), and rural and nonteaching urban hospitals (vs. urban teaching) were associated with fewer diagnoses and procedures, indicating that those hospitals may treat less severe patients and have less available services and resources. Black and Asian patients compared to Whites were associated with more diagnoses or procedures. Black communities often encounter unique barriers to healthcare, including limited access to services, lower rates of health insurance coverage, and cultural factors influencing healthcare-seeking behaviors. These barriers can result in delayed or inadequate healthcare utilization of ambulatory care, leading to increased likelihood of receiving diagnoses or undergoing procedures for certain health conditions at hospitals [17,19]. Medicaid and self-pay hospitalizations were associated with elevated diagnosis counts, possibly reflecting access barriers. These findings underscore the complex association of palliative care utilization as well as sociodemographic, clinical, and hospital-related factors with the numbers of diagnoses and procedures for pediatric patients with neurological and neuromuscular disorders, emphasizing the need for targeted interventions to mitigate disparities in this area.

Hospital LOS and charges also displayed similar, but sometimes different, associations with palliative care utilization, as well as sociodemographic, clinical, and hospital-related factors. Palliative care was a significant contributor to both prolonged length of hospital stay and higher charges, indicative of the complex needs of such patients. This is consistent with some of the literature [32] but differs from what was usually reported for adult patients [19,25] and pediatric palliative care in intensive care units [31,33], which might be related to the reluctance of parents to stop pointless hospital services for their children with neurological and neuromuscular disorders that may not be even verbal to express their sufferings. Age inversely affected hospital LOS and charges, which might be related to very severe conditions of some very young children such as infants and those younger than one year old, their clinical conditions may be harder to stabilize and be more resource-intensive, which needs to be explored further in the future. Black and Asian patients experienced longer LOS and higher charges compared to Whites, which might be related to the higher numbers of diagnoses and procedures observed in the current study. The association of Black and Asian patients with longer LOS and higher charges is consistent with what is observed in the care of other cancer patients [19,21]. Clinical severity significantly prolonged LOS and elevated charges, emphasizing the impact of disease complexity on healthcare resource utilization.

Our findings reveal a significant gap in the utilization of palliative care among pediatric patients with neurological and neuromuscular illnesses, with only a small proportion of pediatric patients receiving these services during inpatient treatment. Factors such as race/ethnicity, severity of illness, insurance type, and hospital characteristics were associated with the palliative care utilization. Pediatric patients receiving palliative care tend to have more complex medical profiles, including a higher number of diagnoses and procedures and more severe illnesses, longer hospital stays, and higher hospital charges. In this study, the opportunities of utilizing palliative care’s benefits, as well as advancement of care efficiency by reducing LOS and total charges, were not lost, as seen in the previous inpatient palliative care study on pediatric cancer cohorts [22]. These findings in this study reveal real-world practice patterns, a potentially late referral from life-sustaining measures to palliative care. This indicates that palliative care is being used for advanced pediatric patients with neurologic and neuromuscular illnesses in hospitals, although it may be recommended that it be offered earlier at ambulatory care settings, if appropriate [19,25,34].

Our findings point to potential disparities in the use of palliative care based on sociodemographic factors (age and sex), clinical characteristics (severity of illness), and hospital features (bed size and teaching status). These patterns highlight the need for targeted strategies to ensure optimal integration of palliative care within hospital settings. The results also carry important policy implications, particularly for improving access to palliative care among pediatric patients with neurologic and neuromuscular conditions. Although palliative care utilization increased over time, suggesting progress in its integration into standard pediatric practice, uptake remains well below that observed in adult populations. This gap indicates that current efforts, while meaningful, are not sufficient on their own. Initiatives such as the Education in Palliative and End-of-Life Care (EPEC)-Pediatrics curriculum, introduced in 2011, have demonstrated improvements in clinicians’ knowledge, attitudes, and skills, as well as in clinical outcomes related to pediatric palliative and end-of-life care. However, broader and more sustained implementation strategies are likely needed to translate these gains into consistently higher utilization across diverse care settings [35]. These pediatric workforce enhancement initiatives are urgently needed and would benefit from a more interprofessional educational approach to strengthen training effectiveness and help reduce disparities associated with low utilization of palliative care in this population.

Second, the observed association between illness severity and use of palliative care highlights the need for targeted strategies to ensure that patients at earlier stages of disease also receive these services, thereby optimizing symptom control and improving quality of life regardless of disease progression [36]. The higher likelihood of palliative care use among patients nearing end of life further suggests that it is often introduced too late and could be integrated earlier to enhance symptom management, pain control, and overall patient well-being [23,35]. Third, addressing the influence of hospital characteristics on palliative care utilization underscores the need for system-level interventions, including more equitable resource allocation and targeted workforce training—particularly in clarifying the distinctions between hospice and palliative care [35,37]—can help strengthen palliative care delivery across hospitals of varying sizes and teaching statuses.

By addressing these policy considerations, healthcare systems can move toward ensuring that all pediatric patients with neurologic and neuromuscular disorders have access to high-quality, patient-centered palliative care that aligns with their individual needs and preferences.

The study had limitations. The retrospective design of the study may introduce biases and limitations associated with relying on medical records for data extraction, such as incomplete documentation or coding inaccuracies [17,19]. Secondly, the study’s focus on palliative care utilization within the hospital setting may overlook palliative care services provided in other settings, such as home or community-based care, which could impact the overall understanding of palliative care utilization among pediatric patients with these conditions [6]. Third, the NIS data does not allow us to directly assess such issues as early versus late in admission, early or late care referral, and patient and family preferences or attitudes towards palliative care limits the depth of understanding regarding factors influencing palliative care utilization in this population. Fourth, patients with multiple hospitalizations during the study period were counted multiple times given that the NIS is a hospital discharge-based dataset. Lastly, no information on hospital palliative care capacity/availability is available in NIS, which makes it impossible to distinguish access/supply issues from referral practices.

Nevertheless, this study’s strengths include being the first research to examine inpatient palliative care among children with neurologic and neuromuscular conditions, based on a large national inpatient database across multiple years, enabling examination of temporal trends and hospital-level variation, and a comprehensive analytical framework integrating sociodemographic, severity of clinical conditions, and hospital characteristics to assess disparities in palliative care utilization at the inpatient setting.

In conclusion, this study offers important insights into the current landscape of inpatient palliative care for pediatric patients with neurologic and neuromuscular conditions at a national level. By identifying factors associated with palliative care utilization and related clinical outcomes, the findings of the study provide a forum for policymakers, clinicians, and researchers to have dialogs about strengthening pediatric palliative care services and improving the overall standard of care for this vulnerable population. Future research may also investigate use of palliative care between pediatric patients who died in the hospital and those who did not, from which how palliative care is offered to children at inpatient settings can be more clearly understood.

## Figures and Tables

**Table 1:** Descriptive results of inpatient palliative care for pediatric neurologic and neuromuscular illnesses (2016–2020, NIS *).

Characteristics	Did Not Receive Palliative Care	Received Palliative Care
N	135,325	4.273
Year (%)		
2016	96.9	3.1
2017	97.2	2.8
2018	97.1	2.9
2019	97.0	3.0
2020	96.3	3.7
Patient characteristics		
Age (year, mean, std.)	6.4 (5.8)	5.3 (5.8)
Sex (%)		
Male	53.8	53.3
Female	46.1	46.6
Race/ethnicity (%)		
White	50.7	48.3
Black	17.0	18.0
Hispanic	21.9	22.4
Asian	3.5	3.8
Native Americans	0.9	1.0
Others	5.9	6.4
Health insurance status (%)		
Private insurance	57.2	61.0
Medicaid	36.0	31.4
Free care	5.2	2.3
Uninsured	1.6	5.3
Median household income by patient ZIP Code (%)		
0–25th percentile	29.5	32.0
26th–50th percentile (median)	25.9	28.2
51st–75th percentile	24.2	22.4
76th–100th percentile	20.2	17.1
Hospitalization		
Numbers of diagnoses (mean, std.)	10.9 (6.5)	17.8 (6.7)
Numbers of procedures received (mean, std.)	2.4 (3.4)	4.0 (4.7)
APR-DRG-Severity (scales 1–4)		
Average scale (mean, std.)	2.6 (0.9)	3.4 (0.7)
1	11.5	3.6
2	30.1	8.2
3	39.4	33.0
4	18.9	55.0
Length of stay (mean, days, std.)	9.91 (21.6)	18.7 (31.4)
Total hospital charges (mean, $, std.)	150,727 (393,098)	332,559 (636,961)
In-hospital death (%)	2.4	32.9
Hospital characteristics		
Hospital bed size (%)		
Small	18.5	16.4
Medium	23.5	20.0
Large	57.9	63.5
Teaching or rural hospital (%)		
Urban nonteaching	95.3	97.4
Urban teaching	3.5	1.8
Rural	1.1	0.7

NIS * = National Inpatient Sample; std = standard deviation.

**Table 2:** Factors associated with palliative care utilization among pediatric patients with neurological and neuromuscular disorders in U.S. hospitals (NIS ^a^, 2016–2020).

Factors	OR ^b^	95% Confidence Intervals	*p*-Value
Year	1.08	[1.05, 1.11]	<0.0001
Age	0.98	[0.97, 0.98]	<0.0001
Gender			
Male (ref)			
Female	1.10	[1.03, 1.18]	0.0052
Race/ethnicity			
White (ref)			
Black	0.90	[0.81, 1.00]	0.0254
Hispanic	0.99	[0.90, 1.09]	0.6111
Asian	1.07	[0.89, 1.29]	0.4598
Native Americans	1.02	[0.72, 1.44]	0.9663
Health insurance			
Private insurance (ref)			
Medicaid	1.07	[0.99, 1.16]	0.6963
Free care	1.22	[0.95, 1.56]	0.1224
Uninsured	0.95	[0.80, 1.12]	0.1027
APR-DRG_Severity ^c^	2.09	[1.99, 2.20]	<0.0001
Median household income by ZIP Code			
76th–100th percentile (ref)			
0–25th percentile	1.09	[0.98, 1.22]	0.4793
26th–50th percentile (median)	1.16	[1.04, 1.29]	0.0093
51st–75th percentile	1.04	[0.93, 1.16]	0.3373
Hospital bed size			
Large (ref)			
Small	0.77	[0.70, 0.85]	0.0016
Medium	0.82	[0.75, 0.90]	0.1423
Hospital teaching and rural status			
Urban, teaching (ref)			
Rural	0.96	[0.65, 1.40]	0.8269
Urban, nonteaching	0.54	[0.42, 0.69]	<0.0001

NIS ^a^ = National Inpatient Sample; ^b^ OR = odds ratio; ^c^ APR-DRG = All-Patient Refined Diagnosis-Related Group.

**Table 3:** Factors associated with the total number of diagnoses and procedures among pediatric patients with neurological and neuromuscular disorders in U.S. hospitals (NIS ^a^, 2016–2020).

Parameter	Number of Diagnoses			Number of Procedures		
	β-Coefficient	SE ^b^	*p*-Value	β-Coefficient	SE ^b^	*p*-Value
Year	0.58	0.01	<0.0001	0.09	0.01	<0.0001
Palliative care	3.42	0.10	<0.0001	0.05	0.07	0.5014
Age	0.02	0.00	<0.0001	−0.04	0.002	<0.0001
Gender						
Male (ref)						
Female	−0.08	0.03	0.0046	−0.08	0.01	<0.0001
Race/ethnicity						
White (ref)						
Black	0.34	0.04	<0.0001	0.18	0.03	<0.0001
Hispanic	−0.03	0.04	0.4776	−0.01	0.02	0.6862
Asian	0.24	0.08	0.0023	0.18	0.05	0.0005
Native Americans	−0.15	0.15	0.2903	−0.22	0.09	0.0139
Health insurance						
Private insurance (ref)						
Medicaid	0.63	0.03	<0.0001	−0.27	0.02	<0.0001
Free care	−0.07	0.11	0.5027	−0.25	0.06	0.0002
Uninsured	0.51	0.07	<0.0001	0.07	0.04	0.1174
APR-DRG_Severity ^c^	4.24	0.02	<0.0001	1.15	0.01	<0.0001
Median household income by ZIP Code						
76th–100th percentile (ref)						
0–25th percentile	0.13	0.05	0.0035	0.05	0.03	0.0751
26th–50th percentile	0.11	0.04	0.0129	−0.03	0.03	0.2411
51st–75th percentile	0.09	0.04	0.0449	−0.03	0.03	0.2741
Hospital Bed size						
Large (ref)						
Small	−0.28	0.04	<0.0001	0.14	0.03	<0.0001
Medium	0.16	0.04	<0.0001	0.14	0.02	<0.0001
Hospital status						
Urban, teaching (ref)						
Rural	−1.22	0.11	<0.0001	−0.96	0.06	<0.0001
Urban, nonteaching	−0.40	0.07	<0.0001	−0.43	0.04	<0.0001

^a^ NIS = National Inpatient Sample; ^b^ SE = standard error; ^c^ APR-DRG = All-Patient Refined Diagnosis-Related Group.

**Table 4:** Factors associated with hospital length of stay and total charges among pediatric patients with neurological and neuromuscular disorders in U.S. hospitals (NIS ^a^, 2016–2020).

Parameter	Length of Stay			Total Charges		
	β-Coefficient	SE ^b^	*p*-Value	β-Coefficient	SE ^b^	*p*-Value
Year	0.51	0.04	<0.0001	9212	810	<0.0001
Palliative care	4.03	0.52	<0.0001	57,000	10,741	<0.0001
Age	−0.62	0.01	<0.0001	−6873	205	<0.0001
Gender						
Male (ref)						
Female	0.01	0.11	0.9240	−1742	2155	0.4189
Race/ethnicity						
White (ref)						
Black	2.32	0.19	<0.0001	28,226	3280	<0.0001
Hispanic	0.09	0.15	0.5708	13,731	3112	<0.0001
Asian	1.50	0.34	<0.0001	19,965	6752	0.0031
Native Americans	0.14	0.62	0.8225	−17,780	9994	0.0752
Health insurance						
Private insurance (ref)						
Medicaid	−0.38	0.13	0.0041	−18,495	2520	<0.0001
Free care	−1.21	0.39	0.0020	−24,834	7822	0.0015
Uninsured	−1.17	0.23	<0.0001	1167	5602	0.8350
APR-DRG_Severity ^c^	7.38	0.08	<0.0001	122,122	1614	<0.0001
Median household income by ZIP Code						
76th–100th percentile (ref)						
0–25th percentile	0.75	0.17	<0.0001	−136	3480	0.9688
26–50th percentile	0.33	0.17	0.0503	−2473	3356	0.4612
51–75th percentile	0.51	0.17	0.0023	−617	3379	0.8551
Hospital Bed size						
Large (ref)						
Small	−0.09	0.16	0.5829	−637	2673	0.8116
Medium	0.38	0.15	0.0137	17,763	3093	<0.0001
Hospital status						
Urban, teaching (ref)						
Rural	−2.11	0.29	<0.0001	−48,953	3308	<0.0001
Urban, nonteaching	1.85	0.35	<0.0001	5980	5681	0.2925

^a^ NIS = National Inpatient Sample; ^b^ SE = standard error; ^c^ APR-DRG = All-Patient Refined Diagnosis-Related Group.

## Data Availability

Data can be purchased from the Agency for Healthcare Research and Quality (AHRQ).

## Supplementary Materials

The following supporting information can be downloaded at: https://www.mdpi.com/article/10.3390/children13081072/s1. File S1: ICD-10 Diagnosis and Procedure Codes for chronic pediatric Neurologic and Neuromuscular conditions.
