# Supplementary material for: Inpatient Palliative Care for Pediatric Neurologic and Neuromuscular Illnesses in the U.S.—A National Perspective
Source: Children (Basel). 2026 Aug 13;13(8):1072. doi: 10.3390/children13081072 (PMC13510643; doi:10.3390/children13081072)
Supplement: Supplementary file 1 [file children-13-01072-s001.zip › children-4180717-supplementary.pdf]

ICD-10 Diagnosis and Procedure Codes for chronic pediatric Neurologic and Neuromuscular conditions

Categories	Subcategories	ICD-10
**Neurologic and Neuromuscular**	Brain and spinal cord malformations	‘Q00-Q07’,‘G901’
	Mental retardation	‘F71-F73’
	CNS degeneration and diseases	‘E750’,’E751’,‘E752’,‘E754’,‘F842’,‘G111,G114’,‘G118’,‘G119’,‘G120’,G122’,‘G128’,‘G129’,‘G3101’,‘G3109’,‘G318’,‘G3189’,‘G3289’,‘G938’,‘G939’,‘G94’,‘G911’,‘G319’,‘G253’,‘G9519’,‘G9589’,‘G909’,‘Q851’
	Infantile cerebral palsy	‘G80’
	Epilepsy	‘G40311’,’G40301’,’G40211’,‘G40219’,‘G40411’,‘G40419’,‘G40111’,‘G40119’,‘G40804’,‘G40911’,‘G40919
	Other disorders of CNS	‘G371’,‘G372’,‘G378’,‘G8190’,‘G8290’,‘G8250-G8254’,‘G835’,‘G839’,‘G931’,‘G935’,‘R403’,‘0016070’,‘0016071’,‘0016072’,‘0016073’,‘0016074’,‘0016075’,‘0016076’,‘0016077’,‘0016078’,‘001607B’,‘0016370’,‘0016371’,‘0016372’,‘0016373’,‘0016374’,‘0016375’,‘0016376’,‘0016377’,‘0016378’,‘001637B’,‘001U074’,‘001U076’,‘001U077’,‘001U079’,‘001U374’,‘001U376’,‘001U377’,‘001U379’,‘00B70ZZ’,‘00B73ZZ’,‘00B74ZZ’,‘00T70ZZ’,‘00T73ZZ’,‘00T74ZZ’
	Occlusion of cerebral arteries	‘I6330’,‘I6350’
	Muscular dystrophies and myopathies	‘G71’,‘G72’
	Movement diseases	‘G10’,‘G20’,‘G210’,‘G2111’,‘G2119’,‘G218’,‘G230-G232’,‘G238’,‘G2402’,‘G248’,‘G253-G255’,‘G2581-G2583’,‘G2589’,‘G259’,‘G803’
	Devices	‘T8509XA’,‘T85190A’,‘T85192A’,‘T85199A’,‘T8579XA’,‘Z982’,‘Z4541’,‘Z4542’,‘00160J0’,‘00160J1’,‘00160J2’,‘00160J3’,‘00160J4’,‘00160J5’,‘00160J6’,‘00160J7’,‘00160J8’,‘00160JB’,‘00160K0’,‘00160K1’,‘00160K2’,‘00160K3’,‘00160K4’,‘00160K5’,‘00160K6’,‘00160K7’,‘00160K8’,‘00160KB’,‘00163J0’,‘00163J1’,‘00163J2’,‘00163J3’,‘00163J4’,‘00163J5’,‘00163J6’,‘00163J7’,‘00163J8’,‘00163JB’,‘00163K0’,‘00163K1’,‘00163K2’,‘00163K3’,‘00163K4’,‘00163K5’,‘00163K6’,‘00163K7’,‘00163K8’,‘00163KB’,‘001U0J4’,‘001U0J6’,‘001U0J7’,‘001U0J9’,‘001U0K4’,‘001U0K6’,‘001U0K7’,‘001U0K9’,‘001U3J4’,‘001U3J6’,‘001U3J7’,‘001U3J9’,‘001U3K4’,‘001U3K6’,‘001U3K7’,‘001U3K9’,‘009600Z’,‘009630Z’,‘009640Z’,‘00H00MZ’,‘00H03MZ’,‘00H04MZ’,‘00H60MZ’,‘00H63MZ’,‘00H64MZ’,‘00HE0MZ’,‘00HE3MZ’,‘00HE4MZ’,‘00HU0MZ’,‘00HU3MZ’,‘00HU4MZ’,‘00HV0MZ’,‘00HV3MZ’,‘00HV4MZ’,‘00W60JZ’,‘00W63JZ’,‘00W64JZ’,‘00WU0JZ’,‘00WU3JZ’,‘00WU4JZ’,‘01HY0MZ’,‘01HY3MZ’,‘01HY4MZ’,‘0DH60MZ’,‘0DH63MZ’,‘0DH64MZ’,‘0W110J9’,‘0W110JB’,‘0W110JG’,‘0W110JJ’,‘3E1Q38X’,‘3E1Q38Z’
